# Supplementary material for: Systems thinking and complexity science methods and the policy process in non-communicable disease prevention: a systematic scoping review protocol
Source: BMJ Open. 2021 Sep 2;11(9):e049878. doi: 10.1136/bmjopen-2021-049878 (PMC8413942; doi:10.1136/bmjopen-2021-049878)
Supplement: Supplementary data [file bmjopen-2021-049878supp001.pdf]

Supplementary file 1

Academic database search strings

We will systematically search electronic databases (Medline, Scopus, Web of Science, EMBASE). The search strategy will be built around four themes representative of the boundaries of the scoping review: methods informed by STCS; different types of policy; domains of the policy process; and NCD prevention. Due to the large numbers of irrelevant records returned by incorporating regulation and related words in the search strategy, the search strategy also sought to exclude records related to genetics. Specific terms used were as follows:

Block 1 – Systems thinking and complexity science

"system theory" OR "system thinking" OR "system science" OR "complex system" OR "system model" OR "system dynamics" OR "system approach" OR "system lens" OR "system perspective" OR complexity OR "complexity theory" OR "complexity science" OR "adaptive system" OR "soft system" OR "agent-based model" OR "group model building" OR "concept mapping" OR "system dynamic" OR "network analysis" OR "partial model testing" OR "system heuristics" OR "causal loop diagram" OR "scenario technique" OR cynefin OR "solution focus" OR behavior-over-time OR "discrete event modelling"

Block 2 – Types of policy

policy OR law OR legal OR legislative OR regulation OR regulate OR regulatory OR tariff OR subsidy OR tax OR ban OR "voluntary agreement" OR incentive OR fiscal OR guidelines OR government

Block 3 – Domains of the policy process

Evaluation OR implementation OR facilitation OR "policy development" OR policymaking OR "case study" OR "problem identification" OR "decision-making" OR strategy OR "policy enactment" OR "policy analysis" OR "stakeholder engagement"

Block 4 – NCD prevention

“public health" OR "health promotion" OR "health inequality" OR "health inequity" OR "health behavior" OR "well being" OR wellbeing OR nutrition OR diet OR obesity OR "fast food" OR “junk food” OR sugar OR salt OR tobacco OR smoking OR cigarette OR alcohol OR "illegal drug*” OR "illicit drug” OR "recreational drug" OR "social determinant" OR “physical activity” OR exercise OR “non-communicable disease” OR “noncommunicable disease” OR “chronic disease” OR “sedentary behaviour” OR NCD

NOT Block 5 – Genetics

Gene OR genetic OR transcript OR transcription OR cell OR nucleus OR mouse OR mice
