# Supplementary material for: Systems thinking and complexity science methods and the policy process in non-communicable disease prevention: a systematic scoping review protocol
Source: BMJ Open. 2021 Sep 2;11(9):e049878. doi: 10.1136/bmjopen-2021-049878 (PMC8413942; doi:10.1136/bmjopen-2021-049878)
Supplement: Author's manuscript [file bmjopen-2021-049878.draft_revisions.pdf]

BMJ Open is committed to open peer review. As part of this commitment we make the peer review history of every article we publish publicly available.

When an article is published we post the peer reviewers' comments and the authors' responses online. We also post the versions of the paper that were used during peer review. These are the versions that the peer review comments apply to.

The versions of the paper that follow are the versions that were submitted during the peer review process. They are not the versions of record or the final published versions. They should not be cited or distributed as the published version of this manuscript.

BMJ Open is an open access journal and the full, final, typeset and author-corrected version of record of the manuscript is available on our site with no access controls, subscription charges or pay-per-view fees (http://bmjopen.bmj.com).

If you have any questions on BMJ Open's open peer review process please email info.bmjopen@bmj.com

BMJ Open

Systems thinking and complexity science methods and the policy process in non-communicable disease prevention: a systematic scoping review protocol

Journal:	*BMJ Open*
Manuscript ID	bmjopen-2021-049878
Article Type:	Protocol
Date Submitted by the Author:	15-Feb-2021
Complete List of Authors:	Clifford Astbury, Chloe; York University Faculty of Health, School of Global Health<br>McGill, Elizabeth; London School of Hygiene & Tropical Medicine, Health Services Research and Policy<br>Egan, Matt; LSHTM, SEHR<br>Penney, Tarra; York University Faculty of Health, School of Global Health
Keywords:	Health policy < HEALTH SERVICES ADMINISTRATION & MANAGEMENT, PUBLIC HEALTH, STATISTICS & RESEARCH METHODS

SCHOLARONE™
Manuscripts

I, the Submitting Author has the right to grant and does grant on behalf of all authors of the Work (as defined in the below author licence), an exclusive licence and/or a non-exclusive licence for contributions from authors who are: i) UK Crown employees; ii) where BMJ has agreed a CC-BY licence shall apply, and/or iii) in accordance with the terms applicable for US Federal Government officers or employees acting as part of their official duties; on a worldwide, perpetual, irrevocable, royalty-free basis to BMJ Publishing Group Ltd ("BMJ") its licensees and where the relevant Journal is co-owned by BMJ to the co-owners of the Journal, to publish the Work in this journal and any other BMJ products and to exploit all rights, as set out in our licence.

The Submitting Author accepts and understands that any supply made under these terms is made by BMJ to the Submitting Author unless you are acting as an employee on behalf of your employer or a postgraduate student of an affiliated institution which is paying any applicable article publishing charge ("APC") for Open Access articles. Where the Submitting Author wishes to make the Work available on an Open Access basis (and intends to pay the relevant APC), the terms of reuse of such Open Access shall be governed by a Creative Commons licence – details of these licences and which Creative Commons licence will apply to this Work are set out in our licence referred to above.

Other than as permitted in any relevant BMJ Author's Self Archiving Policies, I confirm this Work has not been accepted for publication elsewhere, is not being considered for publication elsewhere and does not duplicate material already published. I confirm all authors consent to publication of this Work and authorise the granting of this licence.

For peer review only

Systems thinking and complexity science methods and the policy process in non-communicable disease prevention: a systematic scoping review protocol

Chloe Clifford Astbury, School of Global Health, York University, 4700 Keele St. Toronto ON Canada M3J 1P3 castbury@yorku.ca (corresponding author)

Elizabeth McGill, Department of Health Services Research and Policy, London School of Hygiene and Tropical Medicine, elizabeth.mcgill@lshtm.ac.uk

Matt Egan, Department of Health Services Research and Policy, London School of Hygiene and Tropical Medicine, matt.egan@lshtm.ac.uk

Tarra L. Penney, School of Global Health, York University, Toronto, tpenney@yorku.ca

Abstract

Introduction

Given the complex causal origins of many non-communicable diseases (NCDs), and the complex landscapes in which policies designed to tackle them are made and unfold, the need for systems thinking and complexity science (STCS) in developing effective policy solutions has been emphasised. While numerous methods informed by STCS have been applied to the policy process in NCD prevention, these applications have not been systematically catalogued. The aim of this review is to identify existing applications of methods informed by STCS to the policy process for NCD prevention, documenting which domains of the policy process they have been applied to.

Methods and analysis

A systematic scoping review methodology will be used. Identification: We will search Medline, SCOPUS, Embase and Web of Science using search terms combining STCS, NCD

For peer review only

prevention and the policy process. All records published in English will be eligible for inclusion, regardless of study design. Selection: We will screen titles and abstracts and extract data according to published guidelines for scoping reviews. In order to determine the quality of the included studies, we will use the approach developed by Dixon-Woods and colleagues, excluding studies identified as fatally flawed, and determining the credibility and contribution of included studies. Synthesis: We will summarise key data from each study, mapping applications of methods informed by STCS to different parts of the policy process. This scoping review will identify existing applications of methods informed by STCS to the policy process. Review findings will provide a useful reference for policymakers, outlining which domains of the policy process different methods have been applied to.

Ethics and dissemination

Formal ethical approval is not required, as the study does not involve primary data collection. The findings of this study will be disseminated through a peer-reviewed publication, presentations, and summaries for key stakeholders.

Strengths and limitations of this study

- This scoping review protocol outlines the first piece of work to systematically identify and review how methods informed by systems thinking and complexity science (STCS) have been applied to NCD prevention policy.
- We use the Preferred Reporting Items for Systematic Reviews and Meta-Analyses Protocol checklist (PRISMA-P 2015) in reporting the systematic identification, screening, eligibility of included literature.
- This study will search journals from multiple disciplines to provide a more comprehensive picture of how STCS methods have been applied.
- This scoping review may miss studies that do not self-identify or use the language of methods informed by STCS.

For peer review only - http://bmjopen.bmj.com/site/about/guidelines.xhtml

Introduction

Given the complex and inter-related causes of many non-communicable diseases (NCDs), and the complex realities in which policies designed to tackle them are made and unfold, the need for a 'whole-systems' approach to NCD prevention which encompasses complexity is increasingly recognised (1). Systems thinking and complexity science (STCS) represent a multi-disciplinary field of established and emergent theories and methods (2) which may be usefully applied to NCD prevention. While some approaches to STCS can be expensive and require a high level of technical expertise, others may have value for local and national actors with limited time and resource. It does not need to be costly to think about policies and interventions from a systems perspective. However, there is a need for practical guidance to support such use.

While a distinction has been drawn between systems thinking and complexity science as distinct traditions (3), STCS ideas and methods are broadly characterised by the idea that real-world phenomena exist within systems composed of dynamic actors, including people, organisations and other structures, which evolve in response to each other and their contexts (2). Health systems can be understood in this way, as can other systems with direct health impacts such as transport systems and food systems.

How are systems thinking and complexity science used in public health and health policy?

Acknowledging that phenomena are part of complex systems has repercussions for methods and practice. Methods informed by STCS have been applied to various processes and by varied disciplines, and health researchers have explored their utility in solving seemingly intractable public health issues. These applications are growing rapidly, with as many as 90% of published examples appearing in the past decade (4).

For peer review only

A number of reviews have documented existing approaches to applying STCS to methods and practice in public health and health policy, with most commenting on the relative paucity of studies documenting practical applications of such methods (2,5–7). In the discipline of public health, methods informed by STCS, including systems dynamics modelling, agent-based modelling, soft systems modelling, causal loop diagrams and social network analysis, have been applied (2,7).

While examples of practical applications are limited, where methods informed by STCS have been applied to health policy a number of benefits have been highlighted. A 2015 review focused on the application of a specific method, system dynamics modelling, in support of health policy at any level of government reported that one of the methods key strengths was facilitating consensus-building among stakeholders (5). This was achieved by inviting their participation in developing a model, resulting in agreement over the optimal policy strategy to tackle a given health problem (5).

A 2019 review focusing on how complex systems approaches had been applied in the field of mental health found that research and application in this area was more limited than in other fields of health (6). However, the authors commented on the potential applications of such methods to mental health policy, stating that they might be particularly useful in two processes: first, determining the potential impacts of 'distal' policies, where the policy was removed from its potential impacts in terms of time or causality; and second, assessing what conditions might be necessary for a policy to be successful (6).

Applying systems thinking and complexity science to the policy process

While there has been substantial discussion and theoretical development relating to STCS in the policy process, well-documented examples of how STCS approaches can be applied to this arena, particularly at the national level and in a global context, are less common. Although

For peer review only

some examples have been identified, these either do not result from a comprehensive and systematic review of the literature (4,8), or are restricted to a specific method (5).

Further, a gap exists in determining which of these methods are useful and practical for practitioners with different needs and levels of resource, and in making these distinctions legible to potential users. Scholars of complex systems have previously emphasised the importance of increasing the use of methods informed by STCS in public policy processes, and the responsibility held by researchers to effectively translate their knowledge and methods to encourage their adoption in the policy process (8,9). A review of existing practice which documents clear examples of how these methods can be applied in this context, as well as under what conditions a certain approach might be most useful, is an important part of this process of translation.

For this review to be useful to its intended audience, it is important that it takes into consideration the resources available to policymakers in generating evidence, as well as the ways in which evidence is applied in the policy process. Ghaffarzadegan and colleagues highlight that methods used in the policy process must not only lend themselves well to insight generation, but also to being communicated, as decisions in the policy world must often be justified to stakeholders (8). They argue that some STCS-informed methods might lend themselves more easily to this communication process than others, such as group model building, which supports diverse stakeholders in reaching a consensus, or a small systems dynamics model limited to a smaller number of components, making it easier to interpret (8).

Despite the emphasis on evidence-based policy in public health (10), the role of evidence in policymaking remains relatively limited (11), with policymakers often differing with researchers around what sort of evidence is 'good' and 'useful' (12). Further, evidence generated by researchers may only be inputted at particular points in the policy process, with many parts of this process being a complex series of negotiations between different

For peer review only

perspectives and interests. Given that policymakers already operate in a complex and dynamic space, methods informed by STCS which they may apply to different domains of their work may support them in bringing greater rigour and transparency to the process in its entirety, potentially bringing them closer to policies which may be deemed evidence-based. While many working in policy have expressed an interests in the insights yielded by STCS methods, a recent study of policy evaluators concluded that the methods were in limited use, and that the pragmatic framing of these methods should be seen as a priority to ensure their greater penetration into the process (13).

With this in mind, the aim of this work will be to conduct a scoping review to identify examples of methods informed by STCS being applied to the policy process in the field of NCD prevention.

Methods and analysis

We will conduct a systematic scoping review of peer-reviewed literature documenting the application of methods informed by STCS to the policy process in NCD prevention. The scoping review will be conducted in line with guidelines published by Arksey and O'Malley and refined by Levac and colleagues (14–16), which emphasise an iterative approach suited to an exploratory research question.

In line with these guidelines, this review will be conducted in the following domains (14):

1. Identifying the research question
2. Identifying relevant studies
3. Study selection
4. Charting the data
5. Collating, summarising and reporting the results

For peer review only - http://bmjopen.bmj.com/site/about/guidelines.xhtml

Stage 1: Identifying the research question

The aim of this review is to identify and describe applications of methods informed by STCS to the policy process in NCD prevention, providing insight into how these methods have been and can be applied in this context. Informed by this aim, our central research question is:

- How have methods informed by STCS been applied in the policy process in NCD prevention?

Our sub-research questions are:

1. Which domains of the policy process and areas of NCD prevention policy have methods informed by STCS been applied to?
2. Which methods have been applied by policymakers with different needs and resources?

By policy we refer to *public* policy, defined as 'a set of interrelated decisions taken by a political actor or group of actors concerning the selection of goals and the means of achieving them within a specified situation where those decisions should, in principle, be within the power of those actors to achieve' (17). We understand policy as being ultimately in the hands of government, although we recognise that a number of limitations constrain the policy options available to government, including other domestic and international actors (17,18). For the purposes of this review, we extend the definition of government to include supranational governing bodies.

After Howlett and Cashore, we characterise the policy process as one which moves from broader 'goals' to concrete 'means': specific, on-the ground policy measures designed to achieve the stated goals (18). We use the definition of the domains of the policy process developed by the Centers for Disease Control and prevention (CDC) (see Table 1) (19).

Table 1: The domains of the policy process, from *Overview of CDC's policy process (19)*

**Domain**	**Description**
Problem identification	Clarify and frame the problem or issue in terms of the effect on population health
Policy analysis	Identify different policy options to address the problem/issue and use quantitative and qualitative methods to evaluate policy options to determine the most effective, efficient, and feasible option
Strategy and policy development	Identify the strategy for getting the policy adopted and how the policy will operate
Policy enactment	Follow internal or external procedures for getting policy enacted or passed
Policy implementation	Translate the enacted policy into action, monitor uptake, and ensure full implementation

Stage 2: Identifying relevant studies

We will systematically search electronic databases (Medline, Scopus, Web of Science, EMBASE). The search strategy will be informed by the main concepts in our research question using the Population Concept Context (PCC) Framework recommended by the Joanna Briggs Institute for use in scoping reviews (20) (see Table 2).

Table 2: Concepts from the research question used in developing the search strategy according to the PCC Framework

**Population**	Whole population approach to NCD prevention
**Concept**	Methods and approaches informed by systems thinking and complexity science

For peer review only

Context	Policymaking and different domains of the policy process at different levels of government, including local, national and supranational

Stage 3: Study selection

Records identified through the searches will be collated and double screened using the online platform Covidence (21). Studies will be included where they meet all of the following criteria:

1. Primary study from any country or region, available in English;
2. Self-identify as taking an approach informed by STCS;
3. Report empirical findings from a piece of research done during a specific point in the policy process (problem identification, policy analysis, strategy and policy development, policy enactment, policy implementation, evaluation, stakeholder engagement and education); and
4. Focus on a subject related to NCD prevention.

For academic records, titles and abstracts will initially be screened, following by full-text screening. Full-text screening will be undertaken by two independent researchers.

In line with published guidelines, the approach to study selection may be refined iteratively when reviewing articles for inclusion (14–16).

Stage 4: Charting the data

Data charting will be conducted using a data charting form designed to identify the information required to answer the research question and sub-research questions (see Table 3). As recommended, the data charting form will be piloted with five to ten records to ensure that it is consistent with the research question, and the data charting form will be revised iteratively in order to ensure the purpose of the research is being met (14–16). Where the required

For peer review only - http://bmjopen.bmj.com/site/about/guidelines.xhtml

information is not included in a report, we will follow up with named contacts for additional information.

Table 3: Data charting form

**Record**	Title
	Authors/Organisation
	Year
**Application**	Policy process (problem identification, policy analysis, strategy and policy development, policy enactment, policy implementation) (22)
	Rationale for using STCS (if stated)
	Area of NCD prevention (health outcome or risk factor)
	Policy level (local, national, regional, global)
	Stakeholders involved, if any (government, academic, professional, industry, community)
	Project (state if publication was part of a larger project)
	World region
**Method**	Name
	Tool used (if any: software, kit)
	Aim/research question (if stated)

Stage 5: Collating, summarising and reporting the results

We will undertake quality assessment of the including studies using the approach developed by Dixon-Woods and colleagues, excluding studies identified as ‘fatally flawed’ in the first instance, and determining the credibility and contribution of included studies as part of the synthesis of the evidence (23).

We will analyse the extracted data, presenting a numerical summary of the included studies in table form, allowing us to identify intersections between STCS methods, domains of the policy

For peer review only

process and areas of NCD prevention policy. We will also conduct a thematic analysis of the contents of the included articles in order to identify, if possible, what needs these methods have met and the resources they require, and what challenges were encountered in applying the methods.

Patient and public involvement

Patients or the public were not involved in the design, or conduct, or reporting, or dissemination plans of our research.

Strengths and weaknesses of the study

This review will only identify examples of methods which have previously been applied in the policy domain, and where this application has been documented. We hope this will increase the value of our findings for practitioners, but as a result, methods that have not been applied, or only applied in other fields, will not be identified in this review.

Further, studies that do not 'self-define' as using methods informed by STCS will not be included. Anzola and colleagues highlight the existence of 'analogical' uses of terms relating to complexity, where central characteristics of STCS are employed or implicitly referred to without being explicitly linked to the relevant theory and methods (24). In the absence of shared terminology, such usage may be difficult to systematically identify in the literature. However, given that the aim of this review is to identify the applications of specific methods, we hope this will not result in the exclusion of a large number of relevant studies.

Finally, this review will focus on peer-reviewed literature in order to identify the range of specific and distinct methods that are in use. As a result, applications of methods informed by STCS which are documented in the grey literature will not be identified.

Ethics and dissemination

For peer review only - http://bmjopen.bmj.com/site/about/guidelines.xhtml

Formal ethical approval is not required, as the study does not involve primary data collection.

The further involvement of methods informed by STCS in the policy process will support policymakers in developing evidence-based solutions to complex problems that arise in tackling NCDs. This scoping review will identify existing applications of methods informed by STCS to the policy process. Review findings will provide a useful reference for policymakers, outlining which domains of the policy process different methods have been applied to, and highlighting the resources they require and the problems they have addressed. The findings of this study will be disseminated through a peer-reviewed publication, presentations, and summaries for key stakeholders.

List of abbreviations

STCS: Systems thinking and complexity science

NCD: Non-communicable disease

Declarations

Ethics approval and consent to participate

Not required.

Patient consent for publication

Not required.

Availability of data and material

Data sharing not applicable to this article as no datasets were generated or analysed during the current study.

Competing interests

The authors declare that they have no competing interests.

For peer review

For peer review only - http://bmjopen.bmj.com/site/about/guidelines.xhtml

Funding

CCA and TLP acknowledge research support from York University. EM and ME were supported by the National Institute for Health Research (NIHR) School for Public Health Research (SPHR), Grant Reference Number PD-SPH-2015. The views expressed are those of the author(s) and not necessarily those of the NIHR or the Department of Health and Social Care.

Contributors

CCA and TLP conceived and designed the study. CCA prepared the manuscript. All authors contributed significantly to manuscript revisions and have read and approved the final manuscript.

Acknowledgements

Not applicable.

References

1. Lich KH, Ginexi EM, Osgood ND, Mabry PL. A Call to Address Complexity in Prevention Science Research. Prev Sci. 2013 Jun 1;14(3):279–89.

2. Rusoja E, Haynie D, Sievers J, Mustafee N, Nelson F, Reynolds M, et al. Thinking about complexity in health: A systematic review of the key systems thinking and complexity ideas in health. Journal of Evaluation in Clinical Practice. 2018;24(3):600–6.

3. Gates EF. Making sense of the emerging conversation in evaluation about systems thinking and complexity science. Evaluation and Program Planning. 2016 Dec 1;59:62–73.

4. Atkinson J-A, Page A, Prodan A, McDonnell G, Osgood N. Systems modelling tools to support policy and planning. The Lancet. 2018 Mar 24;391(10126):1158–9.

review only

5. Atkinson J-AM, Wells R, Page A, Dominello A, Haines M, Wilson A. Applications of system dynamics modelling to support health policy. Public Health Res Pract. 2015 Jul 9;25(3):e2531531.

6. Langellier BA, Yang Y, Purtle J, Nelson KL, Stankov I, Diez Roux AV. Complex Systems Approaches to Understand Drivers of Mental Health and Inform Mental Health Policy: A Systematic Review. Adm Policy Ment Health. 2019 Mar 1;46(2):128–44.

7. Carey G, Malbon E, Carey N, Joyce A, Crammond B, Carey A. Systems science and systems thinking for public health: a systematic review of the field. BMJ Open. 2015 Dec 1;5(12):e009002.

8. Ghaffarzadegan N, Lyneis J, Richardson GP. How small system dynamics models can help the public policy process. System Dynamics Review. 2011;27(1):22–44.

9. Forrester JW. System dynamics—the next fifty years. System Dynamics Review. 2007;23(2–3):359–70.

10. WHO | World Health Assembly resolution WHA51.12 - Health promotion [Internet]. WHO. World Health Organization; [cited 2020 Sep 23]. Available from: https://www.who.int/healthpromotion/wha51-12/en/

11. Bambra C. The primacy of politics: the rise and fall of evidence-based public health policy? J Public Health (Oxf). 2013 Dec 1;35(4):486–7.

12. Petticrew M, Whitehead M, Macintyre SJ, Graham H, Egan M. Evidence for public health policy on inequalities: 1: The reality according to policymakers. Journal of Epidemiology & Community Health. 2004 Oct 1;58(10):811–6.

For peer review only

13. Barbrook-Johnson P, Proctor A, Giorgi S, Phillipson J. How do policy evaluators understand complexity? Evaluation. 2020 Jul 1;26(3):315–32.

14. Arksey H, O'Malley L. Scoping studies: towards a methodological framework. International Journal of Social Research Methodology. 2005 Feb 1;8(1):19–32.

15. Levac D, Colquhoun H, O'Brien KK. Scoping studies: advancing the methodology. Implementation Sci. 2010 Sep 20;5(1):69.

16. Colquhoun HL, Levac D, O'Brien KK, Straus S, Tricco AC, Perrier L, et al. Scoping reviews: time for clarity in definition, methods, and reporting. Journal of Clinical Epidemiology. 2014 Dec 1;67(12):1291–4.

17. Jenkins WI. Policy Analysis: A Political and Organisational Perspective. M. Robertson; 1978. 278 p.

18. Howlett M, Cashore B. Conceptualizing Public Policy. In: Engeli I, Allison CR, editors. Comparative Policy Studies: Conceptual and Methodological Challenges [Internet]. London: Palgrave Macmillan UK; 2014 [cited 2020 Oct 19]. p. 17–33. (Research Methods Series). Available from: https://doi.org/10.1057/9781137314154_2

19. Centers for Disease Control and Prevention. Overview of CDC's policy process [Internet]. Atlanta, GA: Centers for Disease Control and Prevention, US Department of Health and Human Services; 2012 [cited 2020 Oct 7]. Available from: https://www.cdc.gov/policy/analysis/process/docs/CDCPolicyProcess.pdf

20. Aromataris E, Munn Z, editors. JBI Manual for Evidence Synthesis [Internet]. Joanna Briggs Institute; 2020 [cited 2020 Sep 23]. Available from: https://wiki.jbi.global/display/MANUAL

For peer review only

21. Covidence - Better systematic review management [Internet]. [cited 2020 Jul 17]. Available from: https://www.covidence.org/home

22. Centers for Disease Control and Prevention. Using Evaluation to Inform CDC's Policy Process [Internet]. Atlanta, GA: Centers for Disease Control and Prevention, US Department of Health and Human Services; 2014 [cited 2020 Jul 17]. Available from: https://www.cdc.gov/policy/analysis/process/docs/UsingEvaluationtoInformCDCsPolicyProcess.pdf

23. Dixon-Woods M, Cavers D, Agarwal S, Annandale E, Arthur A, Harvey J, et al. Conducting a critical interpretive synthesis of the literature on access to healthcare by vulnerable groups. BMC Medical Research Methodology. 2006 Jul 26;6(1):35.

24. Anzola D, Barbrook-Johnson P, Cano JI. Self-organization and social science. Computational and Mathematical Organization Theory. 2016 Jun 16;2(23):221–57.

Appendix

Academic database search strings

We will systematically search electronic databases (Medline, Scopus, Web of Science, EMBASE). The search strategy will be built around four themes representative of the boundaries of the scoping review: methods informed by STCS; different types of policy; domains of the policy process; and NCD prevention. Due to the large numbers of irrelevant records returned by incorporating regulation and related words in the search strategy, the search strategy also sought to exclude records related to genetics. Specific terms used were as follows:

Block 1 – Systems thinking and complexity science

For peer review only

"system theory" OR “system thinking” OR "system science" OR “complex system” OR "system model" OR "system dynamics" OR "system approach" OR "system lens" OR "system perspective" OR complexity OR "complexity theory" OR "complexity science" OR “adaptive system” OR “soft system” OR “agent-based model” OR “group model building” OR “concept mapping” OR “system dynamic” OR “network analysis” OR “partial model testing” OR “system heuristics” OR “causal loop diagram” OR “scenario technique” OR cynefin OR “solution focus” OR behavior-over-time OR “discrete event modelling”

Block 2 – Types of policy

policy OR law OR legal OR legislative OR regulation OR regulate OR regulatory OR tariff OR subsidy OR tax OR ban OR “voluntary agreement” OR incentive OR fiscal OR guidelines OR government

Block 3 – Domains of the policy process

Evaluation OR implementation OR facilitation OR “policy development” OR policymaking OR “case study” OR “problem identification” OR “decision-making” OR strategy OR “policy enactment” OR “policy analysis” OR “stakeholder engagement”

Block 4 – NCD prevention

“public health" OR "health promotion" OR "health inequality" OR "health inequity" OR "health behavior" OR "well being" OR wellbeing OR nutrition OR diet OR obesity OR "fast food" OR “junk food” OR sugar OR salt OR tobacco OR smoking OR cigarette OR alcohol OR "illegal drug*” OR "illicit drug” OR "recreational drug" OR "social determinant" OR “physical activity” OR exercise OR “non-communicable disease” OR “noncommunicable disease” OR “chronic disease” OR “sedentary behaviour” OR NCD

NOT Block 5 – Genetics

Gene OR genetic OR transcript OR transcription OR cell OR nucleus OR mouse OR mice

For peer review only

For peer review only - http://bmjopen.bmj.com/site/about/guidelines.xhtml

For peer review only

PRISMA-P 2015 Checklist

This checklist has been adapted for use with systematic review protocol submissions to BioMed Central journals from Table 3 in Moher D et al: Preferred reporting items for systematic review and meta-analysis protocols (PRISMA-P) 2015 statement. *Systematic Reviews* 2015 **4**:1

An Editorial from the Editors-in-Chief of *Systematic Reviews* details why this checklist was adapted - **Moher D, Stewart L & Shekelle P:** Implementing PRISMA-P: recommendations for prospective authors. *Systematic Reviews* 2016 **5**:15

Section/topic	#	Checklist item	Information reported: Yes	Information reported: No	Line number(s)
**ADMINISTRATIVE INFORMATION**					
**Title**					
Identification	1a	Identify the report as a protocol of a systematic review	☒	☐	p.1 l.1-2
Update	1b	If the protocol is for an update of a previous systematic review, identify as such	☐	☒	N/A
**Registration**	2	If registered, provide the name of the registry (e.g., PROSPERO) and registration number in the Abstract	☐	☒	N/A
**Authors**					
Contact	3a	Provide name, institutional affiliation, and e-mail address of all protocol authors; provide physical mailing address of corresponding author	☒	☐	p.1 l.3-10
Contributions	3b	Describe contributions of protocol authors and identify the guarantor of the review	☒	☐	p.17 l.1-3
**Amendments**	4	If the protocol represents an amendment of a previously completed or published protocol, identify as such and list changes; otherwise, state plan for documenting important protocol amendments	☐	☒	N/A
**Support**					
Sources	5a	Indicate sources of financial or other support for the review	☒	☐	p.16 l.11-16
Sponsor	5b	Provide name for the review funder and/or sponsor	☐	☒	N/A – no project funding
Role of sponsor/funder	5c	Describe roles of funder(s), sponsor(s), and/or institution(s), if any, in developing the protocol	☐	☒	N/A
**INTRODUCTION**					
**Rationale**	6	Describe the rationale for the review in the context of what is already known	☒	☐	p.5 l.1-24, p.6 l.1-20

BioMed Central The Open Access Publisher

Section/topic	#	Checklist item	Information reported Yes	Information reported No	Line number(s)
**Objectives**	7	Provide an explicit statement of the question(s) the review will address with reference to participants, interventions, comparators, and outcomes (PICO)	☒	☐	p.7 l.14-22
**METHODS**					
**Eligibility criteria**	8	Specify the study characteristics (e.g., PICO, study design, setting, time frame) and report characteristics (e.g., years considered, language, publication status) to be used as criteria for eligibility for the review	☒	☐	p.11 l.6-13
**Information sources**	9	Describe all intended information sources (e.g., electronic databases, contact with study authors, trial registers, or other grey literature sources) with planned dates of coverage	☒	☐	p.10 l.1-11
**Search strategy**	10	Present draft of search strategy to be used for at least one electronic database, including planned limits, such that it could be repeated	☒	☐	p.21-23
STUDY RECORDS					
Data management	11a	Describe the mechanism(s) that will be used to manage records and data throughout the review	☒	☐	p.11 l.5-6
Selection process	11b	State the process that will be used for selecting studies (e.g., two independent reviewers) through each phase of the review (i.e., screening, eligibility, and inclusion in meta-analysis)	☒	☐	p.12 l.2-7
Data collection process	11c	Describe planned method of extracting data from reports (e.g., piloting forms, done independently, in duplicate), any processes for obtaining and confirming data from investigators	☒	☐	p.12 l.8-15
**Data items**	12	List and define all variables for which data will be sought (e.g., PICO items, funding sources), any pre-planned data assumptions and simplifications	☒	☐	p.13
**Outcomes and prioritization**	13	List and define all outcomes for which data will be sought, including prioritization of main and additional outcomes, with rationale	☐	☒	N/A – scoping review of application of different methods
**Risk of bias in individual studies**	14	Describe anticipated methods for assessing risk of bias of individual studies, including whether this will be done at the outcome or study level, or both; state how this information will be used in data synthesis	☒	☐	p.13 l.3-6
DATA					
**Synthesis**	15a	Describe criteria under which study data will be quantitatively synthesized	☒	☐	p.12 l.8-15, p.13
	15b	If data are appropriate for quantitative synthesis, describe planned summary measures, methods of handling data, and methods of combining data from studies, including any planned exploration of consistency (e.g., I^2, Kendall's tau)	☐	☒	N/A

BioMed Central The Open Access Publisher

Section/topic	#	Checklist item	Information reported		Line number(s)
			Yes	No	
	15c	Describe any proposed additional analyses (e.g., sensitivity or subgroup analyses, meta-regression)	☐	☒	N/A
	15d	If quantitative synthesis is not appropriate, describe the type of summary planned	☒	☐	p.14 l.2-12
**Meta-bias(es)**	16	Specify any planned assessment of meta-bias(es) (e.g., publication bias across studies, selective reporting within studies)	☐	☒	N/A – scoping review
**Confidence in cumulative evidence**	17	Describe how the strength of the body of evidence will be assessed (e.g., GRADE)	☐	☒	N/A – scoping review

For peer review only

BioMed Central The Open Access Publisher

BMJ Open

Systems thinking and complexity science methods and the policy process in non-communicable disease prevention: a systematic scoping review protocol

Journal:	*BMJ Open*
Manuscript ID	bmjopen-2021-049878.R1
Article Type:	Protocol
Date Submitted by the Author:	13-Jul-2021
Complete List of Authors:	Clifford Astbury, Chloe; York University Faculty of Health, School of Global Health<br>McGill, Elizabeth; London School of Hygiene & Tropical Medicine, Health Services Research and Policy<br>Egan, Matt; LSHTM, SEHR<br>Penney, Tarra; York University Faculty of Health, School of Global Health
**Primary Subject Heading**:	Health policy
Secondary Subject Heading:	Research methods, Public health
Keywords:	PUBLIC HEALTH, STATISTICS & RESEARCH METHODS, Health policy < HEALTH SERVICES ADMINISTRATION & MANAGEMENT

SCHOLARONE™
Manuscripts

I, the Submitting Author has the right to grant and does grant on behalf of all authors of the Work (as defined in the below author licence), an exclusive licence and/or a non-exclusive licence for contributions from authors who are: i) UK Crown employees; ii) where BMJ has agreed a CC-BY licence shall apply, and/or iii) in accordance with the terms applicable for US Federal Government officers or employees acting as part of their official duties; on a worldwide, perpetual, irrevocable, royalty-free basis to BMJ Publishing Group Ltd ("BMJ") its licensees and where the relevant Journal is co-owned by BMJ to the co-owners of the Journal, to publish the Work in this journal and any other BMJ products and to exploit all rights, as set out in our licence.

The Submitting Author accepts and understands that any supply made under these terms is made by BMJ to the Submitting Author unless you are acting as an employee on behalf of your employer or a postgraduate student of an affiliated institution which is paying any applicable article publishing charge ("APC") for Open Access articles. Where the Submitting Author wishes to make the Work available on an Open Access basis (and intends to pay the relevant APC), the terms of reuse of such Open Access shall be governed by a Creative Commons licence – details of these licences and which Creative Commons licence will apply to this Work are set out in our licence referred to above.

Other than as permitted in any relevant BMJ Author's Self Archiving Policies, I confirm this Work has not been accepted for publication elsewhere, is not being considered for publication elsewhere and does not duplicate material already published. I confirm all authors consent to publication of this Work and authorise the granting of this licence.

For peer review only

Systems thinking and complexity science methods and the policy process in non-communicable disease prevention: a systematic scoping review protocol

Chloe Clifford Astbury, Global Food Systems and Policy Research, School of Global Health, York University, Toronto, castbury@yorku.ca

Elizabeth McGill, Department of Health Services Research and Policy, London School of Hygiene and Tropical Medicine, elizabeth.mcgill@lshtm.ac.uk

Matt Egan, Department of Health Services Research and Policy, London School of Hygiene and Tropical Medicine, matt.egan@lshtm.ac.uk

Tarra L. Penney, Global Food Systems and Policy Research, School of Global Health, York University, 4700 Keele St. Toronto ON Canada M3J 1P3, tpenney@yorku.ca (corresponding author)

Abstract

Introduction

Given the complex causal origins of many non-communicable diseases (NCDs), and the complex landscapes in which policies designed to tackle them are made and unfold, the need for systems thinking and complexity science (STCS) in developing effective policy solutions has been emphasised. While numerous methods informed by STCS have been applied to the policy process in NCD prevention, these applications have not been systematically catalogued. The aim of this scoping review is to identify existing applications of methods informed by STCS to the policy process for NCD prevention, documenting which domains of the policy process they have been applied to.

Methods and analysis

For peer review only - http://bmjopen.bmj.com/site/about/guidelines.xhtml

A systematic scoping review methodology will be used. Identification: We will search Medline, SCOPUS, Embase and Web of Science using search terms combining STCS, NCD prevention and the policy process. All records published in English will be eligible for inclusion, regardless of study design. Selection: We will screen titles and abstracts and extract data according to published guidelines for scoping reviews. In order to determine the quality of the included studies, we will use the approach developed by Dixon-Woods and colleagues, excluding studies identified as fatally flawed, and determining the credibility and contribution of included studies. Synthesis: We will identify relevant studies, summarising key data from each study and mapping applications of methods informed by STCS to different parts of the policy process. Review findings will provide a useful reference for policymakers, outlining which domains of the policy process different methods have been applied to.

Ethics and dissemination

Formal ethical approval is not required, as the study does not involve primary data collection. The findings of this study will be disseminated through a peer-reviewed publication, presentations, and summaries for key stakeholders.

Strengths and limitations of this study

- This scoping review protocol outlines the first piece of work to systematically identify and review how methods informed by systems thinking and complexity science (STCS) have been applied to NCD prevention policy.
- We use the Preferred Reporting Items for Systematic Reviews and Meta-Analyses Protocol checklist (PRISMA-P 2015) in reporting the systematic identification, screening, eligibility of included literature.
- This study will search journals from multiple disciplines to provide a more comprehensive picture of how STCS methods have been applied.
- This scoping review may miss studies that do not self-identify or use the language of methods informed by STCS.

For peer review only - http://bmjopen.bmj.com/site/about/guidelines.xhtml

Introduction

Given the complex and inter-related causes of many non-communicable diseases (NCDs), and the complex realities in which policies designed to tackle them are made and unfold, the need for a 'system-level' approach to NCD prevention which encompasses complexity is increasingly recognised (1). Systems thinking and complexity science (STCS) represent a multi-disciplinary field of established and emergent theories and methods (2) which may be applied to NCD prevention. While a contrast has been drawn between systems thinking and complexity science as distinct traditions (3), STCS approaches and methods are broadly characterised by the idea that real-world phenomena exist within systems composed of dynamic actors, including people, organisations and other structures, which evolve in response to each other and their contexts (2).

What role can systems thinking and complexity science play in public health and health policy?

Methods informed by STCS have been applied to various phenomenon of interest by a range of disciplines, and health researchers have explored their utility in solving seemingly intractable public health issues. These applications in public health are growing rapidly, with as many as 90% of published examples appearing in the past decade (4). Several reviews have documented existing approaches to applying STCS to methods and practice in public health. These reviews have made a number of contributions to clarifying terminology and theoretical framing of STCS in public health, including developing frameworks to assess and strengthen complex systems for chronic disease prevention (5) and outlining the range of STCS ideas referred to be public health researchers (2). However, most reviews have commented on the relative paucity of studies documenting practical applications of such methods (2,6–8).

Theoretical discussions around how STCS can be usefully applied to understanding and facilitating the policy process have highlighted the existence of a complex 'policy-making

For peer review only

system', where networks of individuals, organisations and interests interact to produce emergent systemic behaviours (9,10). Characteristics of complexity can be identified throughout the policy process: policy decisions are difficult to predict using deterministic models; policy decisions, once taken, may be implemented in dynamic ways adapted to local contexts by different actors; and implemented policies may have different impacts in different contexts (9). However, discussions of STCS and the policy process have also questioned the extent to which STCS presents policymakers with a 'new' way of approaching their work, given that policymakers may already operate with an implicit awareness of the notion of 'complexity', independently of STCS theory (10).

However, STCS-informed methods may have the added value of bringing more robustness to how policymakers engage with the complexity of the policy process, and providing more opportunities to incorporate research evidence. Despite the emphasis on evidence-based policy in public health (11), the role of research evidence in policymaking remains relatively limited (12), with policymakers often differing with researchers around what sort of evidence is 'good' and 'useful' (13). Further, evidence generated by researchers may only be inputted at particular points in the policy process, with many parts of this process being a dynamic series of negotiations between different perspectives and interests. Given that policymakers already operate in a continually evolving space, methods informed by STCS applied to different domains of their work may bring greater rigour and transparency to the process, and ultimately the utilization of evidence to inform policy. While many working in policy have expressed an interest in the promise of STCS methods to enhance policy, a recent study of policy evaluators concluded that the methods were in limited use, and that the pragmatic framing of these methods should be seen as a priority to ensure their greater penetration into the process (14).

While examples of practical applications are limited, studies have demonstrated the benefits of applying methods informed by STCS to the health policy domain. A 2015 review of system

For peer review only

dynamics modelling in support of health policy at any level of government reported that the method's key strengths included facilitating consensus-building among stakeholders and providing policymakers with dynamic, targeted tools to inform their decisions (6). This review also highlighted ways forward for system dynamics modelling in health policy, including more user-friendly software; better communication of the advantages of system dynamics modelling to policymakers; building capacity to enable more widespread use of this type of modelling; and evaluative evidence to illustrate the benefits of the method (6). A 2019 review of system dynamics and agent-based modelling in mental health research, while identifying a limited number of empirical examples, commented on the potential applications of such methods to mental health policy, stating that they might be particularly useful in two processes: first, modelling the impacts of 'distal' policies, where the policy was removed from its potential impacts in terms of time or causality; and second, assessing what conditions might be necessary for a policy to be successful (7). Finally, Johnston et al. developed an STCS-informed framework which they used to assess a number of North American obesity policy documents (15). This framework used the concept of 'leverage points' within systems, which identify different system components as having the potential to create more or less substantial change (16). This analysis highlighted that many recommendations made in obesity policy focus on leverage points with limited potential to provoke substantial, systems-level change (15).

Aims and scope

While there has been substantive discussion and theoretical development relating to STCS in the policy process, well-documented examples of how STCS approaches can be applied in policymaking for NCD prevention are less common. STCS methods may be usefully applied in other areas of public health characterised by complex interactions between multiple stakeholders and domains, as well as other disciplines more broadly. However, policymaking for NCD prevention has characteristics which may make STCS methods particularly useful.

For peer review only - http://bmjopen.bmj.com/site/about/guidelines.xhtml

First, there are commercial actors and interests involved in NCD-related policy, including the tobacco, alcohol, and food and beverage industries. This adds additional complexity to the policy process and makes a case for transparent approaches to incorporating multiple perspectives and forms of evidence in making policy decisions. Second, despite concerted policy efforts to reduce the burden of NCDs, in many contexts progress has been limited, suggesting that a novel approach that encompasses complexity may be useful (17,18).

Although some relevant examples of STCS methods in the policy process have been identified, these either do not result from a comprehensive and systematic review of the literature (4,19), or are restricted to a specific method (6). Other systematic reviews of STCS in public health do not focus specifically on policymaking (2,5,8), which is a specific context and process in which STCS-informed methods may have particular uses and important strengths and limitations.

Further, a gap exists in determining which of these methods are useful and practical for practitioners with different needs and levels of resource, and in making these distinctions accessible to potential users. Scholars of complex systems have previously emphasised the importance of increasing the use of methods informed by STCS in public policy processes, and the responsibility held by researchers to effectively translate their knowledge and methods to encourage their adoption in the policy process (19,20). A review of existing practice which documents clear examples of how these methods can be applied in this context, as well as under what conditions a certain approach might be most useful, is an important part of this process of translation.

While STCS-informed approaches to understanding policy-making have emphasised its non-linearity, linear or cyclical models of the policy process remain in frequent use by policymakers and practitioners (21,22). In order to facilitate the practical use of review findings, we will use a cyclical model of the policy process (developed by the Centers for Disease Control (CDC)

For peer review only

(21)) to structure the process and results of this review. After Howlett and Cashore, we characterise the policy process as one which moves from broader 'goals' to concrete 'means': specific, on-the ground policy measures designed to achieve the stated goals (23). We use the definition of the domains of the policy process developed by the CDC (see Table 1) (21).

Table 1: The domains of the policy process, from *Overview of CDC's policy process (21)*

Domain	Description
Problem identification	Clarify and frame the problem or issue in terms of the effect on population health
Policy analysis	Identify different policy options to address the problem/issue and use quantitative and qualitative methods to evaluate policy options to determine the most effective, efficient, and feasible option
Strategy and policy development	Identify the strategy for getting the policy adopted and how the policy will operate
Policy enactment	Follow internal or external procedures for getting policy enacted or passed
Policy implementation	Translate the enacted policy into action, monitor uptake, and ensure full implementation

Further, for this review to be useful to its intended audience, it is important that it takes into consideration the resources available to policymakers in generating evidence, as well as the ways in which evidence is applied in the policy process. Ghaffarzadegan and colleagues highlight that methods used in the policy process must not only lend themselves well to insight generation, but also to being communicated, as decisions in the policy world must often be justified to stakeholders (19). They argue that some STCS-informed methods might lend themselves more easily to this communication process than others, such as group model building, which supports diverse stakeholders in reaching a consensus, or a small systems dynamics model limited to a smaller number of components, making it easier to interpret (19).

For peer review only

Therefore, the objective of this review will be to systematically identify and summarise existing applications of STCS-informed methods in NCD prevention policy, documenting key methodological elements and identifying which domains of the policy process they have been applied to.

Methods and analysis

We will conduct a systematic scoping review of peer-reviewed literature documenting the application of methods informed by STCS to the policy process in NCD prevention. The scoping review will be conducted in line with guidelines published by Arksey and O'Malley and refined by Levac and colleagues (24–26), which emphasise an iterative approach suited to an exploratory research question.

In line with these guidelines, this review will be conducted in the following domains (24):

1. Identifying the research question
2. Identifying relevant studies
3. Study selection
4. Charting the data
5. Collating, summarising and reporting the results

Stage 1: Identifying the research question

Informed by our study objective, our central research questions are:

1. How have methods informed by STCS been applied in the policy process in NCD prevention? Which domains of the policy process and areas of NCD prevention policy have methods informed by STCS been applied to?
2. What practical considerations, such as advantages, limitations, barriers and facilitators, have been described in applying STCS-informed methods to NCD prevention policy?

For peer review only - http://bmjopen.bmj.com/site/about/guidelines.xhtml

By policy we refer to *public* policy, defined as 'a set of interrelated decisions taken by a political actor or group of actors concerning the selection of goals and the means of achieving them within a specified situation where those decisions should, in principle, be within the power of those actors to achieve' (27). We understand policy as being ultimately in the hands of government, although we recognise that a number of limitations constrain the policy options available to government, including other domestic and international actors (23,27). For the purposes of this review, we extend the definition of government to include supranational governing bodies.

Stage 2: Identifying relevant studies

We will systematically search electronic databases for peer-reviewed literature (Medline, Scopus, Web of Science, EMBASE). This review will focus on peer-reviewed literature in order to identify the range of specific and distinct methods that are in use. As a result, applications of methods informed by STCS which are documented in the grey literature will not be identified.

The search strategy will be informed by the main concepts in our research question using the Population Concept Context (PCC) Framework recommended by the Joanna Briggs Institute for use in scoping reviews (28) (see Table 2; see Supplementary File 1 for detailed search strategy). Search strategies will be developed iteratively, informed by existing systematic reviews focused on related concepts (5,6,29–31) and indicator papers meeting inclusion criteria of which the authors are aware. As initial searches generated numerous records relating to genetics (due to the inclusion of the term 'regulation' along with health-related terms), a block of NOT terms will be added to the search strategy.

Table 2: Concepts from the research question used in developing the search strategy according to the PCC Framework

For peer review only - http://bmjopen.bmj.com/site/about/guidelines.xhtml

**Population**	Whole population approach to NCD prevention
**Concept**	Methods and approaches informed by systems thinking and complexity science
**Context**	Policymaking and different domains of the policy process at different levels of government, including local, national and supranational

Stage 3: Study selection

Records identified through the searches will be collated and double screened using the online platform Covidence (32). Studies will be included where they meet all of the following criteria:

1. Primary study from any country or region, available in English;
2. Self-identify as taking an approach informed by STCS;
3. Report empirical findings from a piece of research done during a specific point in the policy process (problem identification, policy analysis, strategy and policy development, policy enactment, policy implementation, evaluation, stakeholder engagement and education); and
4. Focus on a subject related to NCD prevention.

For academic records, titles and abstracts will initially be screened, followed by full-text screening. Full-text screening will be undertaken by two independent researchers, one of whom will have extensive experience in the area of STCS and NCD prevention (TLP or EM, who have previously authored reviews on related topics (29,33)). In order to facilitate the identification of methods which may not have been identified as STCS methods by previous reviews of the public health literature (2,8,29), but which authors have identified as STCS

For peer review only - http://bmjopen.bmj.com/site/about/guidelines.xhtml

methods, title-abstract screening will take an inclusive approach, and full texts will be screened to identify STCS language used to describe methods. Papers which focus on healthcare or clinical services rather than primary prevention will be excluded. Papers which concern potential risk factors for NCDs but focus on non-NCD outcomes (such as alcohol consumption as a risk factor for road traffic accidents or inter-personal violence) will also be excluded.

In line with published guidelines, the approach to study selection may be refined iteratively when reviewing articles for inclusion (24–26).

Stage 4: Charting the data

Data charting will be conducted using a data charting form designed to identify the information required to answer the research question and sub-research questions (see Table 3). As recommended, the data charting form will be piloted with five to ten records to ensure that it is consistent with the research question, and the data charting form will be revised iteratively in order to ensure the purpose of the research is being met (24–26). Where the required information is not included in a report, we will follow up with named contacts for additional information.

Table 3: Data charting form

**Record**	Title
	Authors/Organisation
	Year
**Application**	Policy process (problem identification, policy analysis, strategy and policy development, policy enactment, policy implementation) (34)
	Rationale for using STCS (if stated)
	Area of NCD prevention (health outcome or risk factor)
	Policy level (local, national, regional, global)

For peer review only - http://bmjopen.bmj.com/site/about/guidelines.xhtml

	Stakeholders involved, if any (government, academic, professional, industry, community)
	Project (state if publication was part of a larger project)
	World region
**Method**	Name
	Tool used (if any: software, kit)
	Aim/research question (if stated)

Stage 5: Collating, summarising and reporting the results

We will undertake quality assessment of the including studies using the approach developed by Dixon-Woods and colleagues, excluding studies identified as 'fatally flawed' in the first instance, and determining the credibility and contribution of included studies as part of the synthesis of the evidence (35).

We will analyse the extracted data, presenting a numerical summary of the included studies in table form, allowing us to identify intersections between STCS methods, domains of the policy process and areas of NCD prevention policy. We will also conduct a thematic analysis of the contents of the included articles in order to identify, if possible, what needs these methods have met and the resources they require, and what challenges were encountered in applying the methods.

Patient and public involvement

Patients or the public were not involved in the design, or conduct, or reporting, or dissemination plans of our research.

Strengths and weaknesses of the study

This review will only identify examples of methods which have previously been applied in the policy domain, and where this application has been documented. We hope this will increase

For peer review only

the value of our findings for practitioners, but as a result, methods that have not been applied, or only applied in other fields, will not be identified in this review.

Further, studies that do not 'self-define' as using methods informed by STCS will not be included. Anzola and colleagues highlight the existence of 'analogical' uses of terms relating to complexity, where central characteristics of STCS are employed or implicitly referred to without being explicitly linked to the relevant theory and methods (36). Narrative reviews have previously identified implicit complexity concepts in the policy literature (9). However, in the absence of shared terminology, such usage may be difficult to systematically identify in the literature given the reliance of the systematic review method on identifying key words and phrases. In order to include as many relevant examples as possible, we will conduct title-abstract screening in an inclusive way, progressing records to full-text screening if there is any uncertainty. Further, our search strategy has been designed to be relatively inclusive, including broad terms related to STCS, such as complexity and system lens or perspective, as well as specific methods previously identified in systematic reviews.

Ethics and dissemination

Formal ethical approval is not required, as the study does not involve primary data collection.

The further involvement of methods informed by STCS in the policy process will support policymakers in developing evidence-based solutions to complex problems that arise in tackling NCDs. This scoping review will identify existing applications of methods informed by STCS to the policy process. Review findings will provide a useful reference for policymakers, outlining which domains of the policy process different methods have been applied to, and highlighting the resources they require and the problems they have addressed. The findings of this study will be disseminated through a peer-reviewed publication, presentations, and summaries for key stakeholders.

For peer review only - http://bmjopen.bmj.com/site/about/guidelines.xhtml

List of abbreviations

STCS: Systems thinking and complexity science

NCD: Non-communicable disease

Declarations

Ethics approval and consent to participate

Not required.

Patient consent for publication

Not required.

Availability of data and material

Data sharing not applicable to this article as no datasets were generated or analysed during the current study.

Competing interests

The authors declare that they have no competing interests.

Funding

CCA and TLP acknowledge internal research support from York University, Toronto, Canada and the WHO European Office for Prevention and Control of Noncommunicable Diseases. EM and ME were supported by the National Institute for Health Research (NIHR) School for Public Health Research (SPHR), Grant Reference Number PD-SPH-2015. The views expressed are those of the author(s) and not necessarily those of the NIHR or the Department of Health and Social Care.

Contributors

CCA and TLP conceived and designed the study. CCA drafted the manuscript. TLP, EM and ME provided critical input on the manuscript and methods, and have read and approved the final manuscript.

Acknowledgements

The authors would like to acknowledge the useful comments and contributions to the scope of the proposed review by members of the WHO European Office for Prevention and Control of Noncommunicable Diseases.

References

1. Lich KH, Ginexi EM, Osgood ND, Mabry PL. A Call to Address Complexity in Prevention Science Research. Prev Sci. 2013 Jun 1;14(3):279–89.

2. Rusoja E, Haynie D, Sievers J, Mustafee N, Nelson F, Reynolds M, et al. Thinking about complexity in health: A systematic review of the key systems thinking and complexity ideas in health. Journal of Evaluation in Clinical Practice. 2018;24(3):600–6.

3. Gates EF. Making sense of the emerging conversation in evaluation about systems thinking and complexity science. Evaluation and Program Planning. 2016 Dec 1;59:62–73.

4. Atkinson J-A, Page A, Prodan A, McDonnell G, Osgood N. Systems modelling tools to support policy and planning. The Lancet. 2018 Mar 24;391(10126):1158–9.

5. Baugh Littlejohns L, Wilson A. Strengthening complex systems for chronic disease prevention: a systematic review. BMC Public Health. 2019 Jun 11;19(1):729.

6. Atkinson J-AM, Wells R, Page A, Dominello A, Haines M, Wilson A. Applications of system dynamics modelling to support health policy. Public Health Res Pract. 2015 Jul 9;25(3):e2531531.

7. Langellier BA, Yang Y, Purtle J, Nelson KL, Stankov I, Diez Roux AV. Complex Systems Approaches to Understand Drivers of Mental Health and Inform Mental Health Policy: A Systematic Review. Adm Policy Ment Health. 2019 Mar 1;46(2):128–44.

8. Carey G, Malbon E, Carey N, Joyce A, Crammond B, Carey A. Systems science and systems thinking for public health: a systematic review of the field. BMJ Open. 2015 Dec 1;5(12):e009002.

9. Cairney P. Complexity Theory in Political Science and Public Policy. Political Studies Review. 2012 Sep 1;10(3):346–58.

10. Cairney P, Geyer R. A critical Discussion of Complexity Theory: How does 'Complexity Thinking' improve our Understanding of Politics and Policymaking? Complexity, Governance & Networks. 2017 Jun 27;3(2):1–11.

For peer review only - http://bmjopen.bmj.com/site/about/guidelines.xhtml

11. WHO | World Health Assembly resolution WHA51.12 - Health promotion [Internet]. WHO. World Health Organization; [cited 2020 Sep 23]. Available from: https://www.who.int/healthpromotion/wha51-12/en/

12. Bambra C. The primacy of politics: the rise and fall of evidence-based public health policy? J Public Health (Oxf). 2013 Dec 1;35(4):486–7.

13. Petticrew M, Whitehead M, Macintyre SJ, Graham H, Egan M. Evidence for public health policy on inequalities: 1: The reality according to policymakers. Journal of Epidemiology & Community Health. 2004 Oct 1;58(10):811–6.

14. Barbrook-Johnson P, Proctor A, Giorgi S, Phillipson J. How do policy evaluators understand complexity? Evaluation. 2020 Jul 1;26(3):315–32.

15. Johnston LM, Matteson CL, Finegood DT. Systems Science and Obesity Policy: A Novel Framework for Analyzing and Rethinking Population-Level Planning. Am J Public Health. 2014 May 15;104(7):1270–8.

16. Meadows DH. Thinking in Systems: A Primer. Chelsea Green Publishing; 2008. 242 p.

17. Bellew W, Smith BJ, Nau T, Lee K, Reece L, Bauman A. Whole of Systems Approaches to Physical Activity Policy and Practice in Australia: The ASAPa Project Overview and Initial Systems Map. Journal of Physical Activity and Health. 2020 Jan 1;17(1):68–73.

18. Nau T, Lee K, Smith BJ, Bellew W, Reece L, Gelius P, et al. Toward Whole-of-System Action to Promote Physical Activity: A Cross-Sectoral Analysis of Physical Activity Policy in Australia. Journal of Physical Activity and Health. 2019 Nov 1;16(11):1029–38.

19. Ghaffarzadegan N, Lyneis J, Richardson GP. How small system dynamics models can help the public policy process. System Dynamics Review. 2011;27(1):22–44.

20. Forrester JW. System dynamics—the next fifty years. System Dynamics Review. 2007;23(2–3):359–70.

21. Centers for Disease Control and Prevention. Overview of CDC's policy process [Internet]. Atlanta, GA: Centers for Disease Control and Prevention, US Department of Health and Human Services; 2012 [cited 2020 Oct 7]. Available from: https://www.cdc.gov/policy/analysis/process/docs/CDCPolicyProcess.pdf

22. World Health Organization. Health Systems Financing: The Path to Universal Coverage. World Health Organization; 2010. 132 p.

23. Howlett M, Cashore B. Conceptualizing Public Policy. In: Engeli I, Allison CR, editors. Comparative Policy Studies: Conceptual and Methodological Challenges [Internet]. London: Palgrave Macmillan UK; 2014 [cited 2020 Oct 19]. p. 17–33. (Research Methods Series). Available from: https://doi.org/10.1057/9781137314154_2

24. Arksey H, O'Malley L. Scoping studies: towards a methodological framework. International Journal of Social Research Methodology. 2005 Feb 1;8(1):19–32.

25. Levac D, Colquhoun H, O'Brien KK. Scoping studies: advancing the methodology. Implementation Sci. 2010 Sep 20;5(1):69.

For peer review only - http://bmjopen.bmj.com/site/about/guidelines.xhtml

26. Colquhoun HL, Levac D, O'Brien KK, Straus S, Tricco AC, Perrier L, et al. Scoping reviews: time for clarity in definition, methods, and reporting. Journal of Clinical Epidemiology. 2014 Dec 1;67(12):1291–4.

27. Jenkins WI. Policy Analysis: A Political and Organisational Perspective. M. Robertson; 1978. 278 p.

28. Aromataris E, Munn Z, editors. JBI Manual for Evidence Synthesis [Internet]. Joanna Briggs Institute; 2020 [cited 2020 Sep 23]. Available from: https://wiki.jbi.global/display/MANUAL

29. McGill E, Er V, Penney T, Egan M, White M, Meier P, et al. Evaluation of public health interventions from a complex systems perspective: A research methods review. Social Science & Medicine. 2021 Mar 1;272:113697.

30. Langellier BA, Bilal U, Montes F, Meisel JD, Cardoso L de O, Hammond RA. Complex Systems Approaches to Diet: A Systematic Review. American Journal of Preventive Medicine. 2019 Aug 1;57(2):273–81.

31. Bagnall A-M, Radley D, Jones R, Gately P, Nobles J, Van Dijk M, et al. Whole systems approaches to obesity and other complex public health challenges: a systematic review. BMC Public Health. 2019 Jan 3;19(1):8.

32. Covidence - Better systematic review management [Internet]. [cited 2020 Jul 17]. Available from: https://www.covidence.org/home

33. McGill E, Marks D, Er V, Penney T, Petticrew M, Egan M. Qualitative process evaluation from a complex systems perspective: A systematic review and framework for public health evaluators. PLOS Medicine. 2020 Nov 2;17(11):e1003368.

34. Centers for Disease Control and Prevention. Using Evaluation to Inform CDC's Policy Process [Internet]. Atlanta, GA: Centers for Disease Control and Prevention, US Department of Health and Human Services; 2014 [cited 2020 Jul 17]. Available from: https://www.cdc.gov/policy/analysis/process/docs/UsingEvaluationtoInformCDCsPolicyProcess.pdf

35. Dixon-Woods M, Cavers D, Agarwal S, Annandale E, Arthur A, Harvey J, et al. Conducting a critical interpretive synthesis of the literature on access to healthcare by vulnerable groups. BMC Medical Research Methodology. 2006 Jul 26;6(1):35.

36. Anzola D, Barbrook-Johnson P, Cano JI. Self-organization and social science. Computational and Mathematical Organization Theory. 2016 Jun 16;2(23):221–57.

For peer review only

Supplementary file 1

Academic database search strings

We will systematically search electronic databases (Medline, Scopus, Web of Science, EMBASE). The search strategy will be built around four themes representative of the boundaries of the scoping review: methods informed by STCS; different types of policy; domains of the policy process; and NCD prevention. Due to the large numbers of irrelevant records returned by incorporating regulation and related words in the search strategy, the search strategy also sought to exclude records related to genetics. Specific terms used were as follows:

Block 1 – Systems thinking and complexity science

"system theory" OR "system thinking" OR "system science" OR "complex system" OR "system model" OR "system dynamics" OR "system approach" OR "system lens" OR "system perspective" OR complexity OR "complexity theory" OR "complexity science" OR "adaptive system" OR "soft system" OR "agent-based model" OR "group model building" OR "concept mapping" OR "system dynamic" OR "network analysis" OR "partial model testing" OR "system heuristics" OR "causal loop diagram" OR "scenario technique" OR cynefin OR "solution focus" OR behavior-over-time OR "discrete event modelling"

Block 2 – Types of policy

policy OR law OR legal OR legislative OR regulation OR regulate OR regulatory OR tariff OR subsidy OR tax OR ban OR "voluntary agreement" OR incentive OR fiscal OR guidelines OR government

Block 3 – Domains of the policy process

Evaluation OR implementation OR facilitation OR "policy development" OR policymaking OR "case study" OR "problem identification" OR "decision-making" OR strategy OR "policy enactment" OR "policy analysis" OR "stakeholder engagement"

Block 4 – NCD prevention

“public health" OR "health promotion" OR "health inequality" OR "health inequity" OR "health behavior" OR "well being" OR wellbeing OR nutrition OR diet OR obesity OR "fast food" OR “junk food” OR sugar OR salt OR tobacco OR smoking OR cigarette OR alcohol OR "illegal drug*” OR "illicit drug” OR "recreational drug" OR "social determinant" OR “physical activity” OR exercise OR “non-communicable disease” OR “noncommunicable disease” OR “chronic disease” OR “sedentary behaviour” OR NCD

NOT Block 5 – Genetics

Gene OR genetic OR transcript OR transcription OR cell OR nucleus OR mouse OR mice

For peer review only

PRISMA-P 2015 Checklist

This checklist has been adapted for use with systematic review protocol submissions to BioMed Central journals from Table 3 in Moher D et al: Preferred reporting items for systematic review and meta-analysis protocols (PRISMA-P) 2015 statement. *Systematic Reviews* 2015 **4**:1

An Editorial from the Editors-in-Chief of *Systematic Reviews* details why this checklist was adapted - **Moher D, Stewart L & Shekelle P:** Implementing PRISMA-P: recommendations for prospective authors. *Systematic Reviews* 2016 **5**:15

Section/topic	#	Checklist item	Information reported Yes	Information reported No	Line number(s)
**ADMINISTRATIVE INFORMATION**					
**Title**					
Identification	1a	Identify the report as a protocol of a systematic review	☒	☐	p.1 l.1-2
Update	1b	If the protocol is for an update of a previous systematic review, identify as such	☐	☒	N/A
**Registration**	2	If registered, provide the name of the registry (e.g., PROSPERO) and registration number in the Abstract	☐	☒	N/A
**Authors**					
Contact	3a	Provide name, institutional affiliation, and e-mail address of all protocol authors; provide physical mailing address of corresponding author	☒	☐	p.1 l.3-11
Contributions	3b	Describe contributions of protocol authors and identify the guarantor of the review	☒	☐	p.14 l. 21-p.15 l.3
**Amendments**	4	If the protocol represents an amendment of a previously completed or published protocol, identify as such and list changes; otherwise, state plan for documenting important protocol amendments	☐	☒	N/A
**Support**					
Sources	5a	Indicate sources of financial or other support for the review	☒	☐	p.14 l.14-20
Sponsor	5b	Provide name for the review funder and/or sponsor	☐	☒	N/A – no project funding
Role of sponsor/funder	5c	Describe roles of funder(s), sponsor(s), and/or institution(s), if any, in developing the protocol	☐	☒	N/A
**INTRODUCTION**					
**Rationale**	6	Describe the rationale for the review in the context of what is already known	☒	☐	p.5 l.19-25, pp. 6-7

BioMed Central The Open Access Publisher

Section/topic	#	Checklist item	Information reported: Yes	Information reported: No	Line number(s)
**Objectives**	7	Provide an explicit statement of the question(s) the review will address with reference to participants, interventions, comparators, and outcomes (PICO)	☒	☐	p.8 l.17-23
**METHODS**					
**Eligibility criteria**	8	Specify the study characteristics (e.g., PICO, study design, setting, time frame) and report characteristics (e.g., years considered, language, publication status) to be used as criteria for eligibility for the review	☒	☐	p.10, p.11 l.1-7
**Information sources**	9	Describe all intended information sources (e.g., electronic databases, contact with study authors, trial registers, or other grey literature sources) with planned dates of coverage	☒	☐	p.9 l.10-14
**Search strategy**	10	Present draft of search strategy to be used for at least one electronic database, including planned limits, such that it could be repeated	☒	☐	p.17-18
STUDY RECORDS					
Data management	11a	Describe the mechanism(s) that will be used to manage records and data throughout the review	☒	☐	p.10 l.3-4
Selection process	11b	State the process that will be used for selecting studies (e.g., two independent reviewers) through each phase of the review (i.e., screening, eligibility, and inclusion in meta-analysis)	☒	☐	p.10 l.12-17, p.11 l.1-7
Data collection process	11c	Describe planned method of extracting data from reports (e.g., piloting forms, done independently, in duplicate), any processes for obtaining and confirming data from investigators	☒	☐	p.11 l.8-17
**Data items**	12	List and define all variables for which data will be sought (e.g., PICO items, funding sources), any pre-planned data assumptions and simplifications	☒	☐	p.11 (table)
**Outcomes and prioritization**	13	List and define all outcomes for which data will be sought, including prioritization of main and additional outcomes, with rationale	☐	☒	N/A – scoping review of application of different methods
**Risk of bias in individual studies**	14	Describe anticipated methods for assessing risk of bias of individual studies, including whether this will be done at the outcome or study level, or both; state how this information will be used in data synthesis	☒	☐	p.12 l.3-6
DATA					
**Synthesis**	15a	Describe criteria under which study data will be quantitatively synthesized	☒	☐	p.12 l.7-12
	15b	If data are appropriate for quantitative synthesis, describe planned summary measures, methods of handling data, and methods of combining data from studies, including any planned exploration of consistency (e.g., I^2, Kendall's tau)	☐	☒	N/A
	15c	Describe any proposed additional analyses (e.g., sensitivity or subgroup analyses, meta-regression)	☐	☒	N/A

BioMed Central The Open Access Publisher

Section/topic	#	Checklist item	Information reported Yes	No	Line number(s)
	15d	If quantitative synthesis is not appropriate, describe the type of summary planned	☒	☐	p.12 l.7-12
**Meta-bias(es)**	16	Specify any planned assessment of meta-bias(es) (e.g., publication bias across studies, selective reporting within studies)	☐	☒	N/A – scoping review
**Confidence in cumulative evidence**	17	Describe how the strength of the body of evidence will be assessed (e.g., GRADE)	☐	☒	N/A – scoping review

For peer review only

BioMed Central
The Open Access Publisher
